# “They made me feel comfortable”: a comparison of methods to measure patient experience in a sexual health clinic

**DOI:** 10.1186/s12913-017-2264-6

**Published:** 2017-05-05

**Authors:** Alison R. Howarth, Sophie Day, Linda Greene, Helen Ward

**Affiliations:** 1 Patient Experience Research Centre, School of Public Health, Imperial College London, St Mary’s Campus, Norfolk Place, London, W2 1PG UK; 2 Jefferiss Wing Centre for Sexual Health, Imperial College Healthcare NHS Trust, Praed Street, London, W2 1NY UK

**Keywords:** Health services research, Service delivery, Patient experience, Research methodology, Qualitative research

## Abstract

**Background:**

High quality sexual health services are needed to improve both individual and public health outcomes. This study set out to explore what is important to patients who visit a sexual health clinic, and examine their understanding of standard survey questions, in order to inform the collection and interpretation of patient experience data that are used to improve services.

**Methods:**

We conducted a cross-sectional, qualitative study. In the first part of the interview, we used “discovery interviews” to explore patients’ experiences of attending a central London walk-in sexual health clinic. In the second part, we asked patients how they would respond to eight standard patient experience survey questions and to provide an explanation for each of their responses. We conducted a thematic analysis of the interview data.

**Results:**

We interviewed seventeen participants (nine women, eight men) of different ethnicities and backgrounds. All interviewees were positive about their experience. They described how staff had made them feel *“comfortable”*, and talked about how staff spent time, listened and did not rush them, despite being a very busy clinic.

In response to the survey questions, fourteen patients rated their as care excellent or very good overall. However, survey questions were interpreted in different ways and were not always easily understood.

**Conclusions:**

The open-ended “discovery interviews” provided new insights into aspects of care that were most valued or could improve. Standard patient experience questions provide a rating but little elucidation of the experiences that lie behind patients’ responses. They do not always measure aspects of care valued by patients or identify areas for improvement. They are not uniformly understood and necessarily collapse a wide range of experiences and views into categories that may seem inappropriate. Qualitative methods have a key role in measuring patient experience and involving patients in service improvement.

## Background

Patient experience of healthcare is a major indicator of quality across the UK National Health Service (NHS) and remains one of the three key domains of quality [1, 2], alongside patient safety and the clinical effectiveness. The importance of understanding how patients experience care is no more evident than in the provision of sexual health services where both individual and public health benefits may be gained from high quality services which improve sexual health outcomes [3]. We set out to examine patients’ experiences of visiting a sexual health clinic and compare quantitative and qualitative methods of data collection for this purpose.

Surveys are often used to measure patient experience. The NHS patient survey programme has implemented numerous surveys since 2002 and collected data from more than 1.6 million patients on their experience of care in a range of services [4]. The NHS has also introduced a ‘friends and family’ test for in-patient, out-patient, Accident and Emergency (A&E), primary care and maternity services with the aim of identifying the best performing services, and improving patient care through competition [5]. While there is currently no standard measure of satisfaction for sexual health services, a national survey of service users might similarly be used to support commissioning, monitoring and delivery of services [6].

At the same time, there has been a drive towards collecting “real-time feedback” at the point of care, which can be used to measure local quality improvements linked to service providers’ income [7]. While surveys are often used for this purpose, *The Frances Inquiry* into failings at one NHS trust, recommended that qualitative information should be included in this process [8].

While surveying patients about their experience of care and looking at trends over time can inform measures to improve services [9], qualitative research produces more detailed data on different areas of care and a range of patient priorities. In-depth interviews, for example, provided important insights into the experience of using sexual health services not identified in patient questionnaires [10]. However, a systematic review of patient satisfaction in sexual health clinics identified only a limited number of qualitative research projects in the UK [11], most of which examined how specific service provision addresses the needs of particular groups.

The current study used in-depth interviews with patients to explore what was important to them when attending a sexual health clinic. We also examined the interpretation of standard patient experience questions used in the local real-time survey to assess what these questions were measuring. The local survey adopted questions which have been used across a range of conditions and services in the national NHS patient survey programme to gather patients’ views of their care. The questions had been used for more than a decade and changed little over this time.

## Methods

### Design

We undertook a cross-sectional, qualitative study using face-to-face, semi-structured interviews with a purposively selected sample of men and women who had used a central London walk-in sexual health clinic. The following description of the study is guided by ‘The Consolidated Criteria for Reporting Qualitative Studies (COREQ) 32 item checklist’ [12] to ensure transparency and aid critical appraisal. Interviewer and interview characteristics are summarised in Table 1.

**Table 1:** Interviewer and interview characteristics

Interviewer	
Which authors conducted the interviews	ARH
Qualifications	MSc, PhD
Occupation at the time of the study	Research Fellow
Gender	Female
Experience and training	MSc in Advanced Social Research Methods (2001); lead researcher on three studies involving mixed quantitative and qualitative research methods (2003–13)
Relationship with participants	No previous relationship
Interviews	
Setting	Private room on hospital premises
Presence of non-participants	No
Duration	Half an hour
Consent	Consent obtained at time of interview
Audiovisual recording	All interviews audio recorded with participant consent
Transcription	Interviews were transcribed by qualified professionals and checked for accuracy by ARH
Transcripts returned	All interviewees offered a copy of transcript

### Recruitment

Eligible participants were men and women aged 18 years and over who attended the clinic during one of four consecutive days in May 2012. Posters were displayed in the clinic and reception staff gave patients a flier and information sheet about the project at registration. Patients who were interested in being interviewed approached the researcher who was on hand in the clinic. The researcher checked eligibility and patients were purposively selected (by gender, age and sexual orientation) to ensure a range of characteristics and experiences. All participants were offered a £15 high street voucher as a small token of thanks. The participant names given below are all pseudonyms. All patients were given an information sheet explaining the research and introducing the researcher, who went through the sheet with each participant and obtained written consent before the interview started.

### Data collection and analysis

Interviews were conducted on hospital premises, at a convenient time which was usually at the end of the patient’s clinic visit. In the first part of the interview, we adopted a “discovery interview” approach based on a pre-tested topic guide which provided an open-ended framework for discussing the patients’ experience of using the clinic.

In the second part, we asked them how they would respond to eight survey questions and explain each of their responses. In common with other NHS services, the hospital trust within which this study was based has been implementing its own real-time patient experience survey across a broad range of services. We explored patients’ understanding of the eight questions from this survey which were also included in the national outpatient survey 2011 and/or as measures in the Commissioning for Quality and Innovation framework for “*improving responsiveness to personal needs of patients*” [7].

The interviews were recorded and transcribed verbatim (with permission) and took about half an hour. They were conducted and analysed by the primary author.

Framework [13] was used to analyse the data. The key stages of this method include identifying key themes from careful reading of the transcripts, coding the interviews using the ethnographic software NVivo, and summarising (or charting) the data. Word count and text search facilities in NVivo were used to explore key themes and a ‘thick description’ [14] is provided in the results with the aim of supporting the conclusions drawn from this study.

### The setting

The study took place in a large, specialist walk-in sexual health clinic in central London which provides services to more than twenty thousand patients a year. It is open from Monday to Friday for patients to walk-in and see a healthcare professional or they can reserve a time slot by text or online.

## Results

### Participants

Seventeen men and women were interviewed, and their characteristics are shown in Table 2. They were broadly representative of the patient population: routinely collected data (from 2010) show that patients using the service include a range of ethnicities (54% white, 17% mixed or other, 6% South Asian, 20% black, 2% Chinese) and more than half (54%) are born outside the United Kingdom. A greater proportion of male patients (55%) are over 29 years old, compared to female patients (40%). One tenth of male patients are gay or bisexual.

**Table 2:** Background characteristics of patients who took part in interviews

Age group and gender by sexual orientation					
Sexual orientation		Age group		Total	
		29 years or less	30 years or more		
Heterosexual	Male	2	3	5	
	Female	5	4	9	
Gay	Male	-	1	1	
	Female	-	-	-	
Bisexual	Male	-	2	2	
	Female	-	-	-	
	Total	7	10	17	
Used clinic before		Booked a slot		Registered with GP	
No	4	No	15	No	2
Yes	13	Yes	2	Yes	15
Work status		Ethnicity		Country of birth	
Employee	7	White	8	UK	8
Self-employed	2	Asian	2	Africa	3
Student	2	Black	6	Asia	1
Unemployed	6	Other	1	Europe	3
Relationship status		Language spoken at home		Post 16 years education	
Single	13	English	12	None	2
Married	2	No English	3	Up to 4 years	4
Living with partner	2	English and other	2	5 years or more	11

### Making patients feel comfortable

The key theme that emerged from talking to patients related to how comfortable they were made to feel at the clinic. They used the word “comfortable” spontaneously and not in response to any of the prompts in the interview. The majority of the patients had used the clinic before. Among the four patients who were using it for the first time, one said that she expected something more modern and sterile, and another that she had expected it to be full of young people but all four were happy with the service (and rated it as excellent overall).

Coming to the clinic could raise concerns about being judged:

*“I say I don’t care but I was standing outside having a cigarette and I was thinking, ‘I hope I don’t see anybody that I know.’ Because I think some people still think, ‘Oh god, they’re going there.’ But it’s better to go there than not to go there”* (Jane, over 29 years)

Some of the men talked about the intensity of the wait and how other men could sometimes make them feel uncomfortable. The discomfort of waiting could be exacerbated by apprehension:

*“the only time I came to hospital if I’m going to A&E or going with someone else, it didn’t feel that bad. It’s just the whole apprehension of the result which makes it seem longer if I’m being honest”* (Ben, over 29 years)

However, most of the patients talked about how comfortable the staff made them feel. Fifteen of the patients used the word “comfortable” a total of 41 times when describing their visit to the clinic and most did so in a positive way (Table 3). Being made to feel comfortable started with the reception staff – they were friendly, polite, welcoming, respectful, non-judgemental – and continued with the approach of their doctors, nurses and health practitioners, who were professional, open, friendly, calm, polite, considerate, respectful, patient, reassuring, supportive and sympathetic:

**Table 3:** Making patients comfortable

• “So for me it is like, I feel really comfortable to come here to be seen by the doctors, because”; “and then, and she was like, she made me feel comfortable to make any questions. It was all right”; “Because they make you feel comfortable, about the service, about to make any questions, or yes”; “is a hospital. So yes, they make you feel really comfortable, actually you don’t really feel that you are in the”; “good, and they are really friendly, and make you feel comfortable. So it is a good clinic, I would recommend,” (female, under 30 years) • “to make things easy. They try to make you feel comfortable. So I don’t know, they’re laughing a lot. They’re really”; “you just wait there. And here, well the setting is comfortable. You have your water, you have a lot of leaflets”; “and he made me feel at ease, made me feel comfortable. Because I was waiting, and the fact I was wait”; “is there, in maximum one hour you’re done. It’s quite comfortable, the settings in the waiting area, inside the consultation room”; “in the waiting area, inside the consultation room, is really comfortable. I must say that most of the staff are nice” (female, under 30 years) • “I don’t know I just find it too, I'm not comfortable with too much eye contact, I am a teacher and”; “explain something to you, and I just, I'm not entirely comfortable with too much eye contact. Because it feels like they”; “ways of explaining things which I could have been more comfortable with” (female, over 29 years) • “sometimes I think it would be, some people might feel comfortable going back to the other, the same person, like the”; “like you can’t, they’re obviously here to make it as comfortable as possible for you. Obviously I don’t know people’s situations” (male, under 30 years) • “everything. When I sat down they just made me feel comfortable straightaway, broke the ice and that was it I was”; “know if I’m thinking of A&E but it was comfortable and very spaced out, it was actually a lot cooler”; “lot cooler than it was outside. So yes it felt comfortable, I just felt at ease do you know what I” (male, over 29 years) • “but she was very comfortable talking to me about very specific things and understood some”; “and I wanted … whereas the guy I felt much more comfortable with, so saying about the risks of HIV transmission if” (male, over 29 years) • “stuff like that. Then examined me so, I felt very comfortable with that doctor. He said he didn’t know what it” (male, over 29 years) • “me, is it me?” kind of thing. And yes, it’s comfortable anyway”; “her stuff and so yes, for me… Left me feeling comfortable with whatever… She seemed quite experienced, so I felt straight”; “simple, it was answered, so it left me again pretty comfortable, so yes”; “doing a good job, saying that the setup… It’s quite comfortable, and once you’re quite comfortable things must seem good or seem must be right, or” (male, over 29 years) • “I’m trying to remember. You know when you feel comfortable once someone’s questioned you or it’s like they’re interested in”; “So, yes I think you feel more you know like comfortable maybe” (female, over 29 years) • “a lot discussion with the doctor, I really felt very comfortable and I felt it was a really individual approach. So” (female, over 29 years) • “Alison: consultation? How did you feel about that? David: I felt comfortable”; “Alison: anything more that you could say about what makes it comfortable for you? David: It’s just relaxed. It’s very friendly. That’s” (male, over 29 years) • “Which makes you feel more comfortable. So that is all right”; “little things, they do go into it. So I feel comfortable that I don’t have to ask them for a bit; “caring tone they use, and they do make you feel comfortable, because if they weren’t you wouldn’t feel comfortable”; “approach it in a good way, to make you feel comfortable” (female, under 30 years) • “I think as long as any doctor makes you feel comfortable, which he did, he made me feel comfortable, his sex didn’t matter” (female, under 30 years) • “was a much more friendly and made you feel more comfortable than what the doctor did. Then he sort of explains”; “I don’t really like taking tablets. But I don’t feel comfortable enough to say to the doctor, ‘Can I wait until’” (female, over 29 years) • “or turn them off. But I thought it was fine, comfortable seating and loads of leaflets with information on like sexually” (male, under 30 years)

*“everything was very open, it was just small talk in between and that. So she made me feel at ease and obviously she sent me into the next room straightaway and then I saw the nurse … introduced himself so it was first name basis”* (Ben, over 29 years)

The issue of doctor-patient matching was raised by one gay male patient who felt more comfortable with a male doctor who he assumed to be gay and by a female patient who did not mind the gender of her doctor.

Patients talked about the individualised care - how staff listened to them, communicated their options, gave full explanations and reassured them:

*“very, very good to be honest with you, yes. There was nothing that they couldn’t answer for me … any questions I had to ask, even something simple, it was answered, so it left me again pretty comfortable”* (Rob, over 29 years)

*“there was a sense of kind of sensitivity and responsiveness from the doctor, to my needs, not only as a patient but as a female. So I found that extremely accommodating”* (Olivia, over 29 years)

Patients’ comfort was also assured by the way that issues of confidentiality and privacy were managed in the service:

*“having used the service before leaves me quite happy to feel everything’s confidential and the system they have in place is good, so it makes me feel safe to use the service”* (Rob, over 29 years)

Patients described how comfortable facilities enabled them to relax. They were generally very impressed with the amount of time they were given, sometimes drawing comparisons with other NHS appointments where staff did not seem to have time to listen or answer questions:

*“and what I have to say is that she didn’t seem to be in a hurry, or anything like, ‘please hurry I have the next customer’. Or something like that. She was really taking her time, and be there for me, and yes that was really nice”* (Chloe, under 30 years)

On the whole, they accepted that there would be a wait during their visit but they valued the walk-in service, understood that this might mean waiting and appreciated that patients were given the time and attention they needed:

*“so it’s been a very quick service considering it’s a walk-in centre. And I've not felt rushed. I've not felt hurried. I've not felt like the services were under pressure. I felt very relaxed. And there’s been a lot of information given at every step”* (Elizabeth, under 30 years)

### Improvements and recommendations

We asked patients how the service could be improved and what they would recommend about it. They provided a range of suggestions for improvement, including practical ideas, such as something to read in the waiting room. A selection of their recommendations is shown in Table 4. The key reason for recommending the clinic related to the staff, as described above, and the value of their discretion. Patients also highlighted the convenience of the walk-in service and results by text.

**Table 4:** Why patients would recommend the clinic

• “you can book your appointment but it’s going to be quick, yes, reasonable quick. And the staff are nice and they really try to protect your privacy. And … you can have your result by text message” (female, under 30 years) • “very professional … really expert staff, who you never kind of feel like, ‘God, how many more patients have we got to see?’ Or anything like that, you never feel rushed. You really feel that they listen to your concerns, take them seriously, and deal with them. And also with any questions or follow up questions you might have, they care about the whole patient, and they kind of take everything into account I think. Which isn’t often the case I have to say” (female, over 30 years) • “all I can say, when I went it was all right for me. The people, the staff were fine. So hopefully the same experience would happen for somebody else, if I recommended them, so they could recommend somebody else” (male, under 30 years) • “it was discreet, the way they move you about and that, it’s discreet … people are friendly as I said even though I was getting the thing straight to the point, it was still put across in a nice way. The staff are good, they know how to talk, made you feel welcome and gave you everything that you needed at the time so yes it was just really, I liked the service” (male, over 29 years) • “everyone’s very gay friendly and that’s really important. There’s no, it’s very non-judgemental. People are very professional and things do run smoothly but the wait time may be a bit unpredictable. I would say to anybody take a half day, you know put half a day aside just in case” (male, over 29 years) • “if I knew someone with a problem I’d tell them, ‘that’s the only place to go. You’re seen and you’re dealt with and you’ll be content at the end’, so yes” (male, over 29 years) • “the service was very fast, what they were doing was full of care, but it was fast, you didn’t really feel that you were waiting. So that is great … an extremely pleasant environment, friendly and accommodating, in terms of timing, fantastic … I was told this was a very good clinic to go to, and you have asked me whether I have had any expectations, and I did, already because somebody told me it is fantastic. And it is, yes, well done” (female, over 29 years) • “first of all I like it that it is a hospital belonging to a university, that is in my head, or in my imagination, that this is like worth more than some random clinic? So that is what I like” (female, under 30 years) • “the fact that it is a walk-in service … which is really good, and the times are from early to late. So it gives it a lot time to just come in and able to get, the peace of mind, and so it is really good” (female, under 30 years) • “it’s very discreet. It’s clean. It’s efficient. It’s quick. And the doctors and nurses, from my experience, are very good” (female, under 30 years) • “they’re thorough and it’s confidential and you don’t have to be embarrassed about anything when you go, you know, when you go there. I’ve even brought people along” (female, over 29 years) • “I’d just say to come down here because it’s quite easy and straight forward, and I’d say based on today’s experience I’d say it is quite efficient, so you’re here an hour and you’re out, which isn’t too bad” (male, under 30 years)	

### Interpretation of real-time survey questions

We asked our interviewees to describe the experiences that they would draw on in order to answer eight standard patient experience survey questions. Participants were given the question and response options but were not prompted to give a specific response option. We present their responses with the full range of response options and distribution of responses from our interview sample in Table 5. Where specific ratings were not provided, the table lists whether the response was generally positive or affirmative.

**Table 5:** Responses given to survey questions by interviewees

Question	Responses given by interviewees	
1. Overall, how would you rate the care you received?	Excellent	8
	Very good	6
	Good	2
	Fair	0
	Poor	0
	Positive (without specific rating)^a^	1
2. Were you given enough privacy when discussing your condition or treatment?	Yes, always	8
	Yes, sometimes	2
	No	0
	Yes (without specific rating)	7
3. Have you been treated with dignity and respect by staff in this department?	Yes, always	10
	Yes, sometimes	1
	No	0
	Yes (without specific rating)	6
4. Were you involved as much as you wanted to be in decisions about your care and treatment?	Yes, definitely	7
	Yes, to some extent	3
	No	1
	Yes (without specific rating)	6
5. How much information about your condition or treatment has been given to you?	Not enough	4
	The right amount	9
	Too much	0
	Positive (without specific rating)	2
	No answer	2
6. Did you find someone on the hospital staff to talk to about your worries and fears?	Yes, definitely	2
	Yes, to some extent	0
	No	3
	I had no worries or fears	3
	Yes (without specific rating)	7
	No answer	2
7. Did the doctor/nurse explain the reasons for any treatment or action in a way that you could understand?	Yes, completely	6
	Yes, to some extent	3
	No	0
	I did not have any treatment or action	3
	Yes (without specific rating)	5
8. In your opinion how clean was the department?	Very clean	8
	Fairly clean	5
	Not very clean	0
	Not at all clean	0
	Clean (without specific rating)	4

^a^Participants were given the question and response options but were not prompted to give a specific response option; this category refers to participants who spoke in positive terms in response to the question but did not provide a specific response

#### Q1: overall, how would you rate the care you received?

All 17 interviewees rated the care positively, eight (47%) saying it was excellent, six (35%) very good, two good and one positive but not giving a specific rating. Interviewees cited a range of factors when deciding which response to pick: Sarah (over 29 years) contrasted this visit (which she rated very good) with previous occasions which were excellent, because the doctor was not being observed before; Daniel (over 29 years) explained that his care was very good but one particular doctor had been excellent. Others had different reasons for opting for very good rather than excellent: Anna (over 29 years) explained that it was because she had wanted more information about contraception, while Ash (under 30 years) said *“the only reason why I wouldn’t say excellent is because of the screens”* (which are supposed to provide information on waiting times but were not working).

#### Q2: were you given enough privacy when discussing your condition or treatment?

Responses to this question were all positive although two interviewees ticked “yes, sometimes” rather than “yes, always”. The patients described how they were given enough privacy during the consultation and examination: the door was always closed and the curtain drawn in the examination room. Liam (under 30 years) commented on the open space at the reception desk which was less private when patients were talking to reception staff.

#### Q3: have you been treated with dignity and respect by staff in this department?

There was a very positive response to this question. It seemed clear that this was something that patients valued. They explained,

*“oh yes definitely. I felt fine with it. I could still walk out with my head held high”* (Ben, over 29 years)

*“yes I’ve been treated with dignity, always, and that’s why I come back”* (Richard, over 29 years)

#### Q4: were you involved as much as you wanted to be in decisions about your care and treatment?

While the overall response was positive, one interviewee gave a negative response, and people were not clear about the meaning of the question. Patients talked about staff listening and responding to them and being provided with the information they needed to make an informed choice (for example, about what tests to have or the method of examination), but some did not see that there was much need for their involvement. They commented on the seamlessness of their appointments:

*“yes, I would say so. I mean, there was no need for me to do anything, but whatever was kind of like going on you were just going with the flow, so ye*s” (Rob, over 29 years)

Some questioned whether they had a role in decisions:

*“I don't know what it means by were you involved as much. I mean it's basically their decision that I should do the test, so you know there’s nothing to be involved with really”* (Daniel, over 29 years)

#### Q5: how much information about your condition or treatment has been given to you?

Most interviewees felt that the information given was about right, although four chose “not enough”: Sophia (under 30 years) said her *condition* had not been explained although she was told that she was fine, Rob (over 29 years) said the information about his *treatment* had been quite basic, while two patients had been given medication but were unclear about their condition because they needed to return for further test results.

#### Q6: did you find someone on the hospital staff to talk to about your worries and fears?

Only two interviewees said yes definitely, three said no but three said that they had no worries or fears. The patients generally felt reassured and able to talk to the doctors and nurses but William (over 29 years) commented that he could not answer the question because he did not need to “*seek out somebody*” as everything was explained to him. The three patients who reported not finding anyone went on to explain: Ben (over 29 years) needed to come back for another appointment so did not ask for anyone; David (over 29 years) did not have any worries or fears, and Ash (under 30 years) referred to when he was told to take a seat after he had registered but did not know what would happen from there.

#### Q7: did the doctor/nurse explain the reasons for any treatment or action in a way that you could understand?

Not everyone found this easy to answer: Fiona (under 30 years) commented on how she never needed to ask staff to explain medical jargon, while two of the patients who were not native English speakers commented on how sensitive staff were to language differences. Rob (over 29 years) felt that the diagnosis was still sinking in when information was being given, while Jane (over 29 years) did not understand why she had been given tablets without a confirmed diagnosis.

#### Q8: in your opinion how clean was the department?

All our interviewees chose “very clean” or “fairly clean”. Patients appreciated that newspapers, cups and rubbish were cleared up.

## Discussion

Our study highlights the key role of qualitative research methods in measuring patient experience and gathering data for service improvement. Our interviews captured the elements of care that patients valued while their responses to standard patient experience questions added little to the understanding of how patients experienced care and what could be done to improve it.

Patients generally express discomfort about attending sexual health services [15] which is characterised by high levels of anxiety and negative preconceptions [10]. This requires management of a number of elements including worries about censure [16], feelings of stigmatisation and embarrassment [17], concerns about confidentiality and privacy [17–19], vulnerability associated with genital examination [19] and fear of the unknown [16]. The patients interviewed for this study described how staff had made them feel *“comfortable”*, spent time with them, listened and did not rush them, even though it is a very busy clinic. Other NHS services may find this relevant to their own situation and future research could be usefully undertaken to better understand what can be done to make patients feel comfortable.

Talking to patients also provides an opportunity for them to suggest improvements. While they were generally very happy with the service, they also made constructive suggestions – such as something to read in the waiting room–which the service was able to address. Asking them about why they would recommend the service, reinforces understanding of aspects of the service that patients value–from results by text to staff discretion – providing important feedback for service improvement.

Standard patient experience questions provide ratings which can be used to compare experiences between services and over time, but they provide little insight into why patients respond in the way that they do. We found that patients’ reasoning behind their answers to the survey questions identified aspects of their experience which may be used for service improvements but which were not elicited in response to the actual questions.

Good survey design requires that questions are understood in the same way by all respondents [20]. Our study suggests variation in this process. Patients drew on different experiences while answering the same question, making it difficult to interpret their answers. A clear example is the question about being involved in decisions about care and treatment. Some patients answered the question about *treatment* which they saw as referring specifically to medical treatment, whereas others talked about involvement in other aspects of *care*. Patients also had different understandings and aspirations in relation to their involvement in decisions or desire for information. The question thus confused people who did not consider that they should be involved in decisions about treatment and found no obvious way of responding.

Patient experience surveys have a key role to play in monitoring services, but such surveys should be tailored to the patient population. While we welcome the development of a questionnaire specifically designed to measure patient satisfaction with sexual health services [6], our study indicates that questions will need careful selection and testing if they are to provide useful data that are valid and reliable.

A limitation of our study is the extent to which the experiences of the patients that we interviewed represent those of the patient population. Our sample was self-selected from among those who attended the walk-in sexual health clinic during one of four consecutive days in May 2012. While the participants came from a range of ethnicities and backgrounds and our findings are congruent with previous studies, our sample is unlikely to have captured the full diversity of the population that use the walk-in sexual health clinic. More heterogeneous samples are likely to report still more varied experiences of care [21], however, suggesting that our findings may underestimate the variation in how patients interpret standard survey questions.

## Conclusions

Our study has demonstrated the importance that people place on being made to feel comfortable when they attend a sexual health clinic. It has also highlighted the role of qualitative research in uncovering this finding and enabling a better understanding of patients’ experiences and needs. The NHS ‘friends and family’ test aims to improve patient care by identifying the best performing hospitals and providing models of service for lower ranking units [5]. Patients are asked if they would recommend hospital wards, for example, to a friend or relative based on their treatment. The question is controversial, partly because of the objection that patients may interpret it in different ways [22]. Our study suggests that the question will need substantial qualitative research of the type reported here if we are to understand *why* patients respond to it in the way they do and gather data that can be used for service improvement.

## Abbreviations

A&E: Accident and Emergency
NHS: National Health Service
